# Kininase 1 As a Preclinical Therapeutic Target for Kinin B_1_ Receptor in Insulin Resistance

**DOI:** 10.3389/fphar.2017.00509

**Published:** 2017-08-02

**Authors:** Youssef Haddad, Réjean Couture

**Affiliations:** Department of Pharmacology and Physiology, Faculty of Medicine, Université de Montréal Montréal, QC, Canada

**Keywords:** bradykinin, carboxypeptidase M, inducible nitric oxide, inflammatory markers, insulin resistance, Mergetpa, oxidative stress

## Abstract

Kinin B1 receptor (B1R) contributes to insulin resistance, an early event in type 2 diabetes, through the upregulation and activation of the inducible form of nitric oxide synthase (iNOS), pro-inflammatory cytokines and the oxidative stress. This study addresses the hypothesis that inhibition of kininase 1 (carboxypeptidase M, CPM), the key enzyme involved in the biosynthesis of B1R agonists, could exert the same beneficial effects to B1R antagonism in insulin resistance. Male Sprague-Dawley rats were made insulin resistant with a drinking solution containing 10% D-glucose for a period of 9 weeks. Control rats received tap water. During the last week, kininase 1 was blocked with Mergetpa (1 mg kg^−1^ twice daily, s.c.) and the impact was determined on insulin resistance (HOMA index), metabolic hormone levels, oxidative stress and the expression of several markers of inflammation by western blot and qRT-PCR. Glucose-fed rats displayed hyperglycemia, hyperinsulinemia, hyperleptinemia, insulin resistance, hypertension, positive body weight gain, and enhanced expression of B1R, CPM, iNOS, and IL-1β in renal cortex, aorta and liver. Markers of oxidative stress (superoxide anion and nitrotyrosine expression) were also enhanced in aorta and renal cortex. Mergetpa reversed and normalized most of those alterations, but failed to affect leptin levels and hypertension. Pharmacological blockade of kininase 1 (CPM) exerted similar beneficial effects to a 1-week treatment with a B1R antagonist (SSR240612) or an iNOS inhibitor (1,400 W). These data reinforce the detrimental role of B1R in insulin resistance and recommend CPM as a new therapeutic target.

## Introduction

Kininase I-type carboxypeptidases are key enzymes involved in the biotransformation of native kinin agonists (bradykinin (BK) and Lys-BK or kallidin) acting at the constitutive B2 receptor (B2R) into B1 receptor agonists (des-Arg^9^-BK and Lys-des-Arg^9^-BK) by specifically removing the COOH-terminal Arg residue (Zhang et al., 2013a). An important constitutively active membrane-bound kininase 1 is carboxypeptidase M (CPM, EC 3.4.17.12), an Arg-carboxypeptidase expressed in a wide variety of cell types, including renal, vascular, neural, pulmonary, and immune cells (Deiteren et al., 2009). This enzyme is strategically localized to regulate kinin activity in inflammatory processes as it is upregulated during tissue damage and by pro-inflammatory cytokines similarly to the B1R (Schremmer-Danninger et al., 1998; Deiteren et al., 2009; Kashuba et al., 2013; Couture et al., 2014). In addition to generating B1R agonists in close proximity to the receptor, biochemical studies in transfected cells suggest that CPM interacts with the B1R to enhance B1R signaling. Kinin (BK or kallidin) binding to the CPM active site causes a conformational activation of the B1R (Zhang et al., 2013a) and basal binding of CPM to extracellular loop 2 of the B1R results in positive allosteric modulation of B1R signaling to its orthosteric agonist (Zhang et al., 2013b). Recently, CPM and B1R were found upregulated along with the inducible nitric oxide synthase (iNOS) and interleukin-1β (IL-1β) in aorta, renal cortex and liver in a rat model of insulin resistance induced by high glucose feeding (Haddad and Couture, 2016). Inhibition of iNOS for 1-week with 1,400 W (N- (3-aminomethyl-benzyl acetamidine) (Haddad and Couture, 2016) or B1R with SSR240612 (Dias et al., 2010; Dias and Couture, 2012a,b) reversed insulin resistance and its associated metabolic features (hyperglycemia, hyperinsulinemia) through the inhibition of the oxidative stress and the nuclear factor NF-κB pathway leading thereby to the concomitant suppression of CPM, B1R, iNOS, and IL-1β overexpression. These studies provided evidence that iNOS and B1R are engaged in a reciprocal upregulation that contributes to insulin resistance and peripheral inflammation.

In addition to upregulating iNOS in insulin resistance, B1R can activate iNOS via Gαi and the Src-dependent activation of the ERK/MAPK pathway leading to a large production of nitric oxide (NO) (Kuhr et al., 2010; Brovkovych et al., 2011). Moreover, B1R stimulation was shown to enhance the production of superoxide anion (O_2_^•−^) through NADPH oxidase in vessel of glucose-fed rats and in human epithelial cells (Dias et al., 2010; Talbot et al., 2011). B1R-induced overproduction of NO and O_2_^•−^ is harmful because both molecules can interact rapidly to form peroxynitrite (ONOO^−^), a highly toxic molecule, which causes DNA damage that alters gene expression, inflammation and oxidative stress, notably lipid peroxidation of membranes and nitration of various proteins (enzymes, transporters, ionic pumps, ion channels) (Johansen et al., 2005; Ascenzi et al., 2010). We reported that prolonged inhibition of iNOS with 1,400 W (Haddad and Couture, 2016) blunted the production of peroxynitrite in the model of insulin resistance, suggesting that this oxidative pathway contributes to the upregulation of the biomarkers of inflammation (iNOS, CPM, B1R, and IL-1β) and represents an important mechanism leading to insulin resistance.

The present study was designed to test the hypothesis that inhibition of CPM with Mergetpa also named Plummer's inhibitor (Plummer and Ryan, 1981; Charest-Morin et al., 2014) is a valid strategy to prevent the biosynthesis of B1R agonists and thereby may offer the same therapeutic effect to B1R antagonists in insulin resistance. Mergetpa can also prevent the biotransformation of plasma kinins by carboxypeptidase N (CPN), the plasma kininase 1 (Erdos and Sloane, 1962; Salgado et al., 1986). However, intriguing reports mentioned that dynorphin A may be an endogenous ligand of BK receptors (Lai et al., 2006, 2008; Lee et al., 2014) while cathepsin X localized at the surface of endothelial cells, in macrophages and monocytes can produce B1R ligands from BK and Lys-BK (Nagler et al., 2010). Thus, the use of Mergetpa should ascertain whether B1R is activated by its classical kinin metabolites des-Arg^9^-BK and Lys-des-Arg^9^-BK derived from kininase 1. Therefore, the impact of a 1-week pharmacological treatment with Mergetpa was determined on insulin resistance, metabolic hormones levels, hypertension, vascular and non-vascular inflammatory markers (iNOS, B1R, CPM, IL-1β) and oxidative stress (O_2_^•−^ and ONOO^−^) in the model of insulin resistance induced by high glucose feeding. This study tends to support the contribution of kininase I and its active classical B1R metabolites in insulin resistance. Because kininase 1 inhibition reproduces the therapeutic effects of B1R antagonists, CPM may represent a new therapeutic target.

## Materials and methods

### Animal care and experimental procedures

All experimental methods and animal care procedures were approved by the Animal Care Committee of the Université de Montréal (protocol 11–140) in accordance with the Canadian Council on Animal Care and in compliance with the ARRIVE guidelines (Kilkenny et al., 2010; McGrath and Lilley, 2015). Male Sprague-Dawley rats (24–30 days old, 50–75 g) were purchased from Charles River Laboratories (St-Constant, QC, Canada) and housed two per cage, under standard conditions (22.5°C and 42.5% humidity, on a 12 h/12 h light-dark cycle), and allowed free access to a standard chow diet (Charles River Rodent) and to a drinking solution containing 10% D-glucose or tap water (control) for a period of 9 weeks.

**Figure 1:**
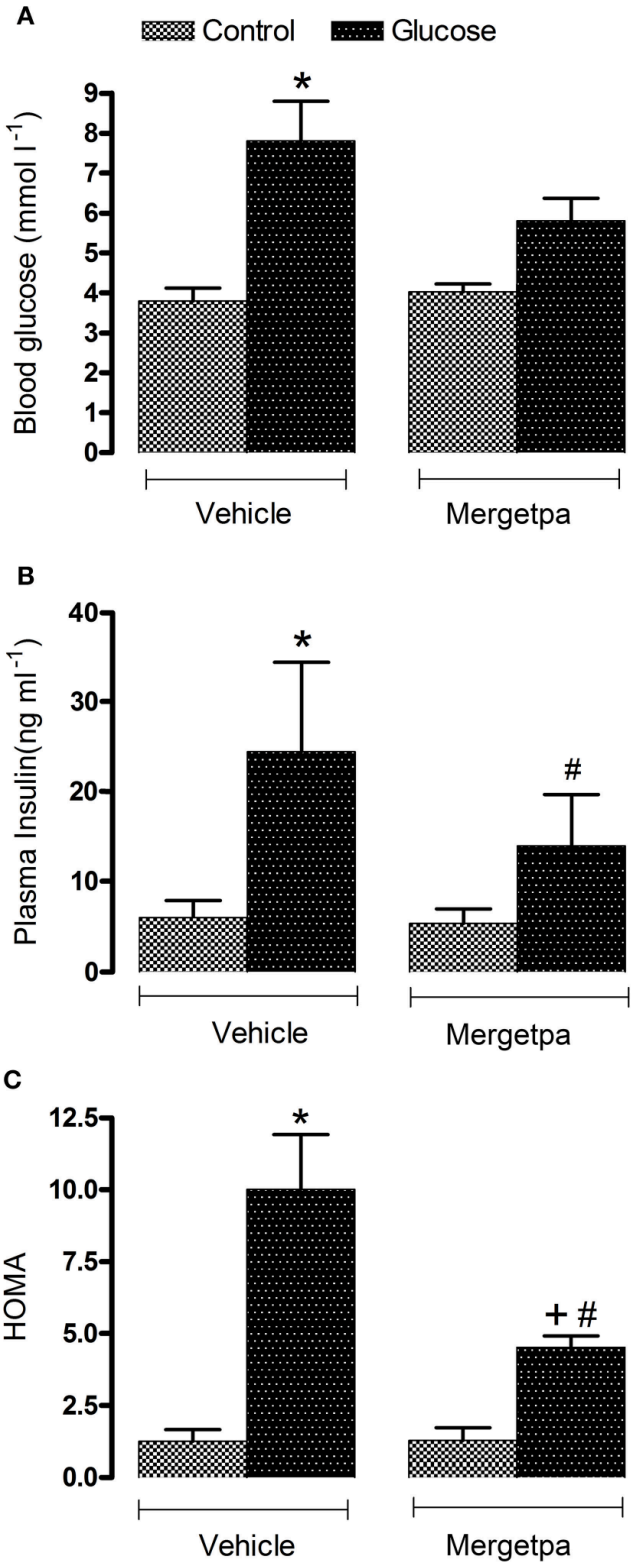
Treatment effect on metabolic parameters. Effect of s.c. administered Mergetpa (1 mg.kg ^−1^ twice daily) for 7 days on **(A)** blood glucose, **(B)** plasma insulin and **(C)** insulin resistance assessed by the HOMA index. Data are mean ± SEM obtained from six rats per group. ^*^P < 0.05 compared with control + vehicle; ^+^*P* < 0.05 compared with glucose + vehicle; ^#^*P* < 0.05 compared with control + Mergetpa. Data in vehicle treated rats are from a previous study (Haddad and Couture, 2016) but performed simultaneously.

**Figure 2:**
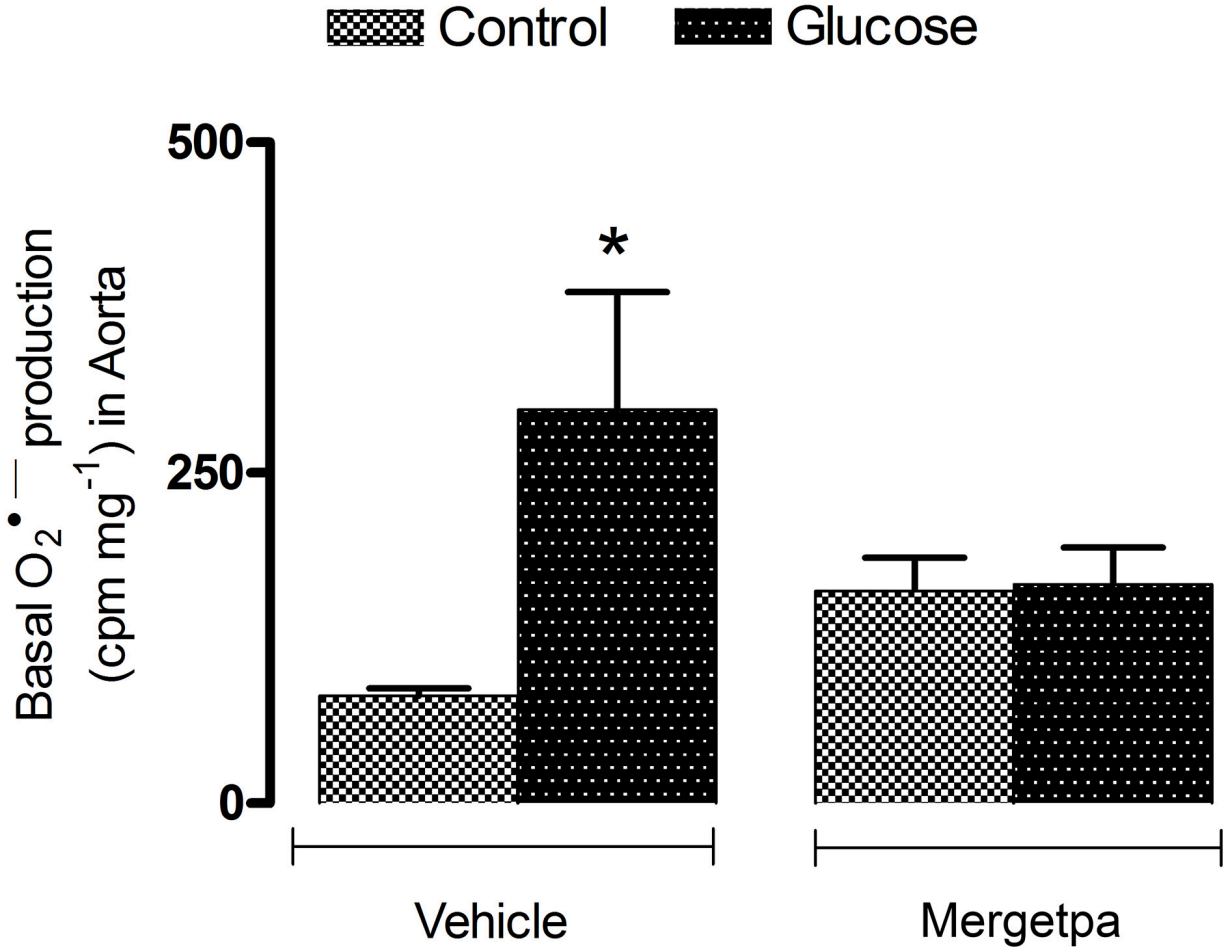
Treatment effect on anion superoxide production. Effect of s.c. administered Mergetpa (1 mg.kg ^−1^ twice daily) for 7 days on basal superoxide anion production in the thoracic aorta. Data are mean ± SEM obtained from six rats per group. ^*^P < 0.05 compared with control + vehicle. Data in vehicle treated rats are from a previous study (Haddad and Couture, 2016) but performed simultaneously.

On the ninth week, rats fed with glucose (G) or tap water (C) were randomly divided into six groups of six rats: group 1 (G + Mergetpa), group 2 (G + Vehicle), group 3 (C + Mergetpa), group 4 (C + Vehicle), group 5 (G + 1,400 W) and group 6 (C + 1,400 W). Data of groups 5 and 6 were published elsewhere (Haddad and Couture, 2016). New data are presented herein with Mergetpa (groups 1 and 3), yet the control groups 2 and 4 served in both studies as they were run in the same series of experiments. Mergetpa (DL-2-Mercaptomethyl-3-guanidinoethylthiopropanoic acid), a high affinity and reversible inhibitor of Arg-carboxypeptidase (CPM/CPN named kininase I) (Plummer and Ryan, 1981; Charest-Morin et al., 2014) was purchased from Calbiochem (La Jolla, CA, USA). It was given s.c. twice daily at 12 h intervals at the dose of 1 mg.kg^−1^ in a volume of 1 mL.kg^−1^ for 7 days. The vehicle (saline) was given in groups 2 and 4. The impact of vehicle (saline) itself was not tested in tap water (C) and glucose (G) groups as key data (Figures 1, 2 and B1R expression) were similar to those obtained earlier in C and G groups not treated with vehicle (Dias et al., 2010). Thus, the effect of Mergetpa was compared to a placebo (vehicle) in both C and G groups. In a rare study using Mergetpa *in vivo*, a bolus dose of 10 mg.kg^−1^ followed by 1 mg.kg^−1^ per min continuous infusion potentiated the hypotensive response to intraarterial injection of BK (Salgado et al., 1986). Thus, we selected the minimal dose of 1 mg.kg^−1^ twice a day. A treatment of 1-week was used to enable better comparison with our earlier studies using 1-week treatment with the iNOS inhibitor 1,400 W and the B1R antagonist SSR240612 (Dias et al., 2010; Dias and Couture, 2012a,b; Haddad and Couture, 2016). During the last week, groups 1, 2, 3, and 4 were subjected to several measurements, including systolic blood pressure, blood glucose and body weight.

At the end of the ninth week, the blood was collected by cardiac puncture in overnight-fasted rats under isoflurane anesthesia. Blood samples were collected in tubes containing anticoagulant (Heparin; Sandoz, Boucherville, QC, Canada), and the plasma was separated by centrifugation at 600 × g for 15 min at 4°C and stored at −20°C. Tissues and organs (thoracic aorta, kidneys, liver) were removed and kept frozen at −80°C. The renal cortex was dissected out just prior to experiment. They were selected to enable comparison with our previous studies carried out with the iNOS inhibitor 1,400 W and the B1R antagonist SSR240612 (Dias et al., 2010; Dias and Couture, 2012a; Haddad and Couture, 2016). Proteins, DNA and RNA of these organs were extracted to measure the expression of several inflammatory biomarkers by western blotting and quantitative RT-PCR (qRT-PCR). Superoxide anion was measured in the aorta using a chemiluminescence technique and lucigenin.

### Measurement of systolic blood pressure

Systolic blood pressure was measured by tail-cuff plethysmography using a pad heated at 37°C under the tail. The values were registered with the system Instruments ADI (ADI Instruments Inc., Colorado, CO, USA) assisted by the ADI Instruments software (Pro7.Ink Chart Lab). Each value corresponded to the average of 5–7 measurements taken at 1–2 min intervals. Rats were familiarized with the tail-cuff system once a day for a week before starting the systolic blood pressure measurements.

### Plasma analysis

Blood glucose concentration was determined with a glucometer (Accu-Chek Aviva; Roche Diagnostics, Laval, QC, Canada). The plasma insulin and leptin concentrations were determined by RIA (rat insulin RIA kit and rat leptin RIA kit) from Millipore (St. Charles, MO, USA). The homeostasis model assessment (HOMA) index was used to assess insulin resistance by calculating the value of fasting insulin and glucose with the following formula: [insulin (μU·mL^−1^) × glucose (mmol·L^−1^)/22.5] (Matthews et al., 1985).

### Measurement of superoxide anion

Superoxide anion (O_2_^•−^) was measured in the thoracic aorta with the chemiluminescence method using lucigenin (Munzel et al., 1995; Haddad and Couture, 2016). Slices of thoracic aorta (2–5 mg) were pre-incubated in a Krebs-HEPES buffer (saturated with 95% O_2_ and 5% CO_2_ at room temperature) for 30 min and transferred to a scintillation vial containing 5 μM of lucigenin to determine the basal level of O_2_^•−^. Chemiluminescence was then recorded every minute for 10 min in a dark room using a liquid scintillation counter (Wallac 1409, Turku, Finland). The background was counted using a vial with no tissue solution. The final value was calculated as follows: (tissue value − background value) ÷ tissue dry weight and was expressed in cpm.mg^−1^ of dry weight tissue.

### Western blot analysis

The western blot was performed as previously described (Pouliot et al., 2011; Haddad and Couture, 2016). Dynein and β-actin were used as standard proteins. A quantitative analysis of proteins was provided by scanning densitometry using the MCID-M1 system (Imaging Research, St. Catharines, ON, Canada).

Detection of B1R was made with a selective antibody (1:1,000) rose in rabbits (Biotechnology Research Institute, Montreal, QC, Canada) against a conserved amino acid sequence from mouse and rat B1R protein. The epitope used contained 15 amino acids localized in the C-terminal part of the B1R (VFAGRLLKTRVLGTL). Care was taken to avoid sequences with similarity to related mammalian proteins, including the opposite B2R. One negative control was run using the pre-immune serum. Specificity of anti-B1R antibody was further determined using mouse kidney extracts from wild-type and B1R knockout (KO) mice (Lin et al., 2010; Lacoste et al., 2013).

The other primary antibodies were as follows: dynein (1:4,000 mouse, SC-13524), β-actin (1:5,000 mouse, SC-47778), IL-1β (1:500 rabbit, SC-7884), iNOS (NOS2) (1:1,000 rabbit, SC-650), carboxypeptidase M (1:500 rabbit, SC 98698) (SC: Santa Cruz Biotechnology, CA, USA), and nitrotyrosine (1:1,500 mouse,1A6-05233; Millipores, Billerica, MA, USA). Secondary antibodies were horseradish peroxidase (HRP)-linked goat anti-rabbit SC-2004 and HRP-linked goat anti-mouse SC-2005 (Santa Cruz) used at dilution of 1:25,000 (for B1), 1:5,000 (for Dynein, β-actin, IL-1β, iNOS, and CPM) and 1:3,000 (nitrotyrosine).

### Quantitative real-time polymerase chain reaction (qRT-PCR)

At sacrifice, around 10 mg of tissue (renal cortex, liver and thoracic aorta) were put in a RNA*later* stabilization reagent (QIAGEN, Toronto, ON, Canada) and frozen at −56°C. Total RNA was extracted from the tissue using Qiazol according to the manufacturer's instructions. The single-stranded cDNA was synthesized according to the procedure in the manual supplied by Bio-Rad. qRT-PCR was performed in the SYBR Green Master Mix (QIAGEN) by adding 300 nM of each primer and the signal was detected by the Mx3000p device (Stratagene, La Jolla, CA, USA) and using rat 18S as standard. The primer pairs were designed by Vector NTI software as documented earlier (Haddad and Couture, 2016). The PCR conditions were: 95°C for 15 min followed by 46 cycles at 94°C for 15 s, 60°C for 30 s and 72°C for 30 s. The cycle threshold value represents the number of cycles during which a fluorescent signal increases above the background noise. The relative quantification of gene expression was analyzed by the method of 2^−ΔΔCt^ (Livak and Schmittgen, 2001).

### Statistical data analysis

Data are presented as mean ± SEM, and *n* represents the number of rats in each group. Statistical analysis was performed using Prism™ version 5.0 (GraphPad Software Inc., La Jolla, CA, USA); data and statistical analysis comply with the recommendations on experimental design and analysis in pharmacology (Curtis et al., 2015). Statistical significance was determined with Student's *t*-test for unpaired samples or with the one-way ANOVA followed by the Bonferroni test for multiple comparisons when F achieved *P* < 0.05 and there was no significant variance in homogeneity. Only the value of *P* ≤ 0.05 was considered to be statistically significant.

## Results

### Effect of Mergetpa on clinical parameters

Blood glucose level was significantly increased by two-fold (*P* < 0.05) in overnight-fasted glucose-fed rats compared with control rats, yet after 1-week treatment with Mergetpa (1 mg kg^−1^ twice daily) glycemia was reduced to level that was no longer significantly different from control values. Plasma insulin level was significantly increased by four-fold (*P* < 0.05) in glucose-fed rats and was halved by the treatment with Mergetpa, but this reduction did not reach significance. The HOMA index of insulin resistance was also significantly enhanced (*P* < 0.05) in glucose-fed rats, yet this value was markedly reduced (*P* < 0.05) but not completely normalized by Mergetpa. In contrast, the same treatment with Mergetpa failed to affect glycemia, insulinemia and the HOMA index in control rats (Figure 1).

The body weight was not significantly different between glucose-fed rats and control rats before (8 weeks) and after (9 weeks) treatment with Mergetpa (Table 1). However, the gain in body weight was significantly higher (*P* < 0.05) in glucose-fed rats after 9 weeks when compared with age-matched controls. The 1-week treatment with Mergetpa had no impact on the gain in body weight in control rats, but a significant loss in body weight gain (*P* < 0.05) was measured after treatment with Mergetpa in glucose-fed rats. The 1-week treatment with Mergetpa had no significant effect on plasma leptin levels in both control and glucose-fed rats, which remained significantly enhanced (*P* < 0.05) in the latter group as compared with control values. Systolic blood pressure was significantly enhanced (*P* < 0.05) in 9-week glucose-fed rats compared with control rats and the high systolic blood pressure value was not significantly affected by the 1-week treatment with Mergetpa (Table 1).

**Table 1:** Body weight, plasma leptin levels and systolic blood pressure.

**Parameters**	**Control + vehicle**	**Glucose + vehicle**	**Control + mergetpa**	**Glucose + mergetpa**
Body weight at the initiation of protocol (g)	114 ± 3	110 ± 3	107 ± 3	111 ± 2
Body weight (g) at 8 weeks	587 ± 25	592 ± 29	538 ± 38	622 ± 29
Body weight (g) at 9 weeks	603 ± 26	635 ± 18	554 ± 38	604 ± 17
Body weight gain/loss (g)	16 ± 2	43 ± 2^*^	16 ± 4	(−)18 ± 2.0^+^^#^
Plasma leptin (ng.ml^−^1)	3.8 ± 0.3	10.4 ± 2.2^*^	4.5 ± 1.4	11.7 ± 2.6^#^
Systolic blood pressure (mm Hg) at 9 weeks	109 ± 3	127 ± 3^*^	119 ± 4	129 ± 6

Values represent the mean ± SEM of six rats per group. Statistical comparison (^*^) to control + vehicle, (+) to glucose + vehicle and (#) to control + Mergetpa is indicated by

* *P < 0.05*,

+ *P < 0.05*,

# *P < 0.05*.

*Data in vehicle treated rats are from a previous study (Haddad and Couture, 2016) but performed simultaneously*.

### Effect of Mergetpa on oxidative stress

The oxidative stress was assessed by the measurement of superoxide anion and the expression of nitrotyrosine, a marker of peroxynitrite (ONOO^−^). The latter molecule causes tyrosine nitration by the covalent addition of NO_2_ to tyrosine residues in various proteins thereby affecting protein function and stability. Data depicted in Figure 2 show that the basal production of superoxide anion was significantly increased (*P* < 0.05) in the aorta of glucose-fed rats when compared with control rats. The 1-week treatment with Mergetpa brought back to control values the enhanced basal production of superoxide anion in the aorta of glucose-fed rats. Mergetpa did not affect significantly the production of superoxide anion in control aorta.

**Figure 3:**
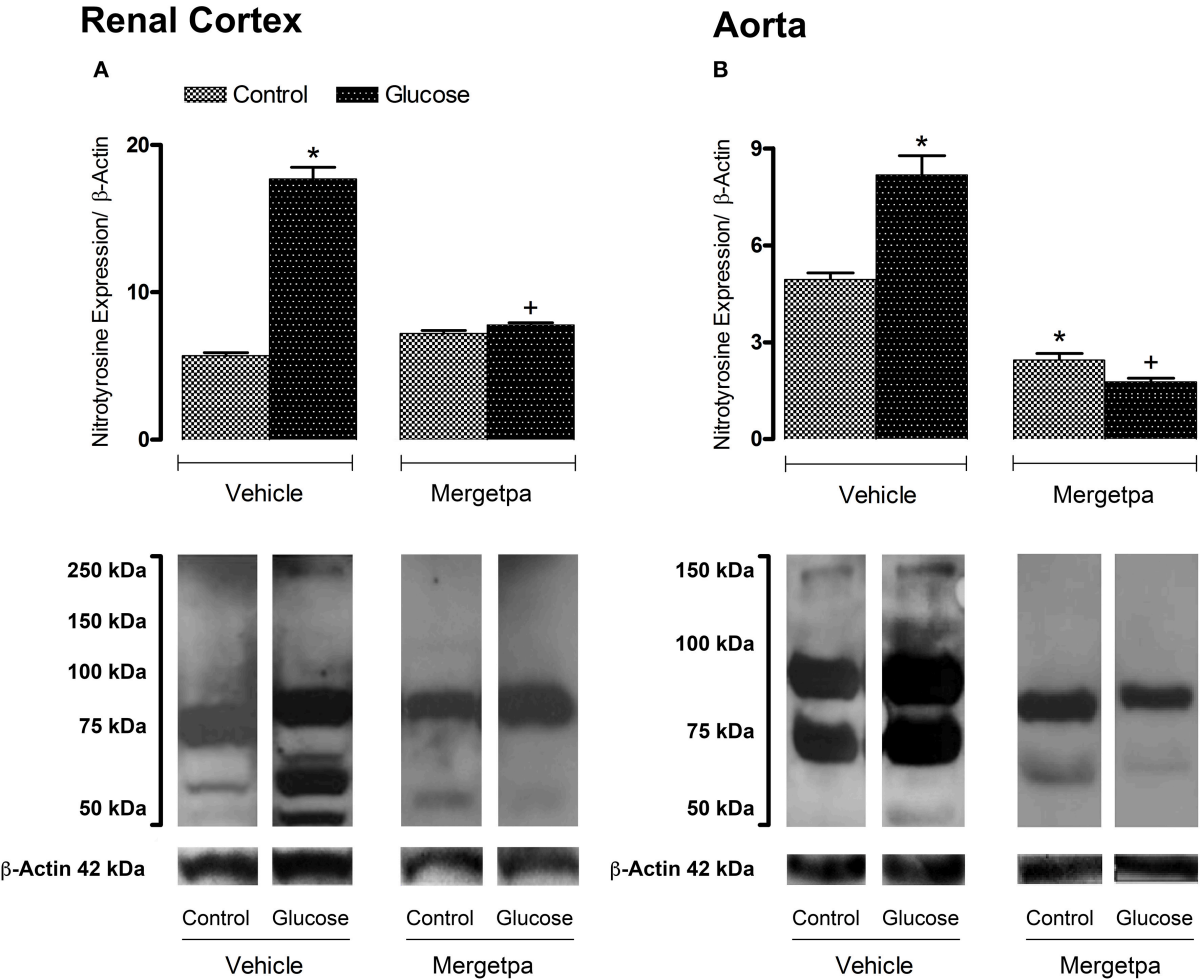
Treatment effect on Nitrotyrosine expression. Effect of s.c. administered Mergetpa (1 mg.kg ^−1^ twice daily) for 7 days on nitrotyrosine expression in **(A)** renal cortex and **(B)** thoracic aorta. β-actin was the loading control. Data are mean ± SEM obtained from six rats per group. ^*^P < 0.05 compared with control + vehicle; ^+^*P* < 0.05 compared with glucose + vehicle. Data in vehicle treated rats are from a previous study (Haddad and Couture, 2016) but performed simultaneously.

Nitrotyrosine expression was markedly enhanced (*P* < 0.05) in the renal cortex (Figure 3A) and aorta (Figure 3B) of glucose-fed rats as visualized by increases in the intensity of nitrotyrosine epitopes and the occurrence of new tyrosine nitration proteins between 50 and 250 kDa (renal cortex) or 50 and 150 kDa (aorta) when compared to control tissues. Treatment with Mergetpa significantly reduced (*P* < 0.05) the intensity of nitrotyrosine-containing proteins in renal cortex and aorta of glucose-fed rats to levels not significantly different from control values. Nitrotyrosine expression was also significantly reduced in control aorta but not in control renal cortex by Mergetpa (Figure 3).

### Effect of Mergetpa on B1R and carboxypeptidase M expression

**Figure 4:**
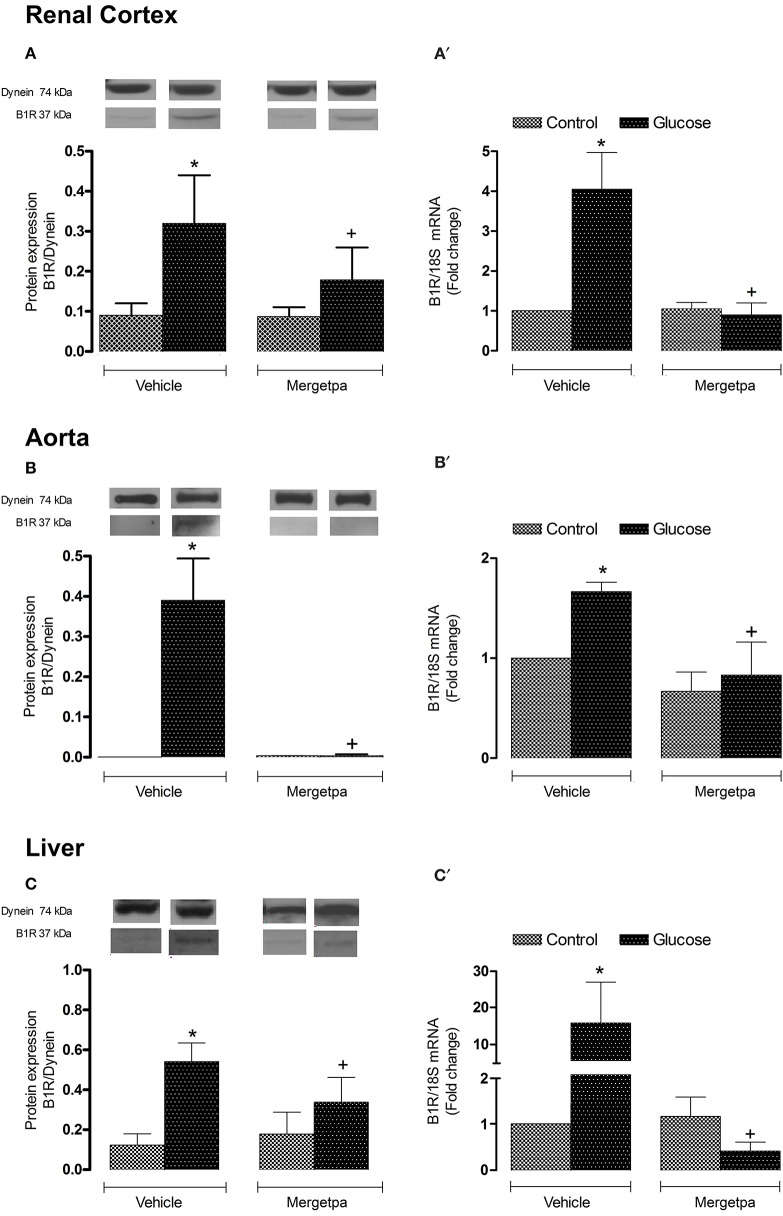
Treatment effect on B1R expression. Effect of s.c. administered Mergetpa (1 mg.kg ^−1^ twice daily) for 7 days on B1R expression in **(A,A')** renal cortex, **(B,B')** thoracic aorta and **(C,C')** liver. The expression of B1R was measured at the protein level by western blot **(A–C)** and at mRNA level by qRT-PCR **(A',B',C')**. Dynein was the loading control. Data are mean ± SEM obtained from six rats per group. ^*^P < 0.05 compared with control + vehicle; ^+^*P* < 0.05 compared with glucose + vehicle. Data in vehicle treated rats are from a previous study (Haddad and Couture, 2016) but performed simultaneously.

Both B1R protein expression and B1R mRNA levels were significantly enhanced (*P* < 0.05) in renal cortex (Figures 4A,A'), thoracic aorta (Figures 4B,B') and liver (Figures 4C,C') of glucose-fed rats. The 1-week treatment with Mergetpa brought back to control levels B1R protein and mRNA expression in the three tissues of glucose-fed rats, but failed to affect B1R protein and mRNA expression in the same tissues of control rats (Figure 4).

**Figure 5:**
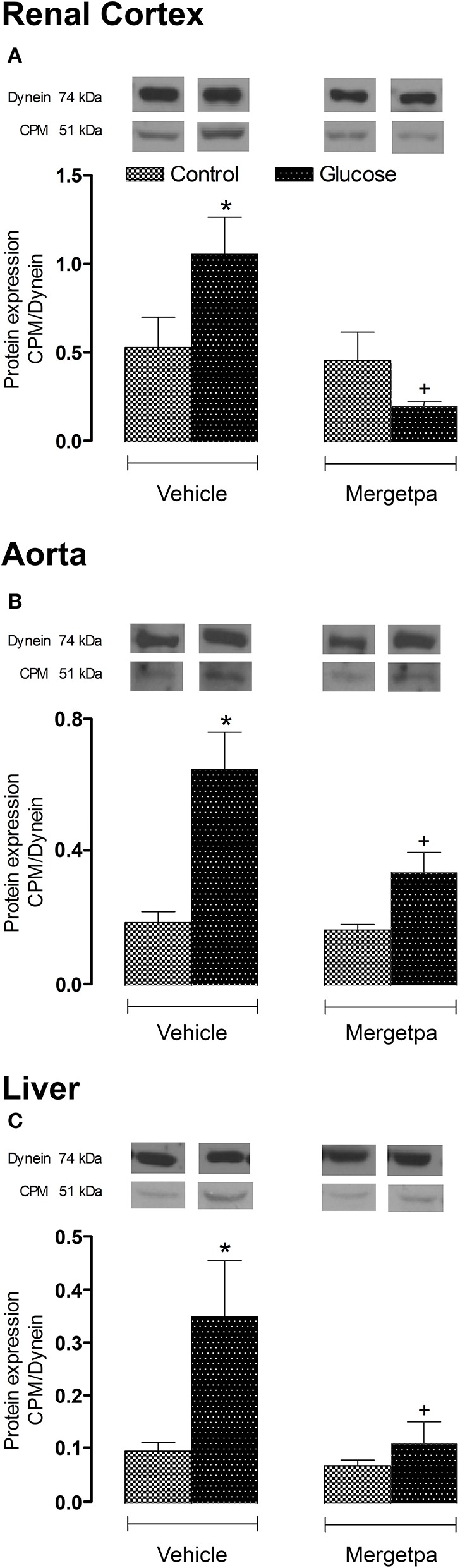
Treatment effect on CPM expression. Effect of s.c. administered Mergetpa (1 mg.kg ^−1^ twice daily) for 7 days on CPM expression in **(A)** renal cortex, **(B)** thoracic aorta, and **(C)** liver. The expression of CPM was measured at the protein level by western blot and dynein was the loading control. Data are mean ± SEM obtained from six rats per group. ^*^P < 0.05 compared with control + vehicle; ^+^*P* < 0.05 compared with glucose + vehicle. Data in vehicle treated rats are from a previous study (Haddad and Couture, 2016) but performed simultaneously.

Likewise to B1R, CPM protein expression was significantly enhanced (*P* < 0.05) in renal cortex, thoracic aorta and liver of glucose-fed rats (Figures 5A–C). Whereas the 1-week treatment with Mergetpa failed to modify CPM protein expression in control tissues, this treatment blocked completely (P < 0.05) the overexpression of CPM in the three studied tissues of glucose-fed rats (Figure 5).

### Effect of Mergetpa on iNOS expression

**Figure 6:**
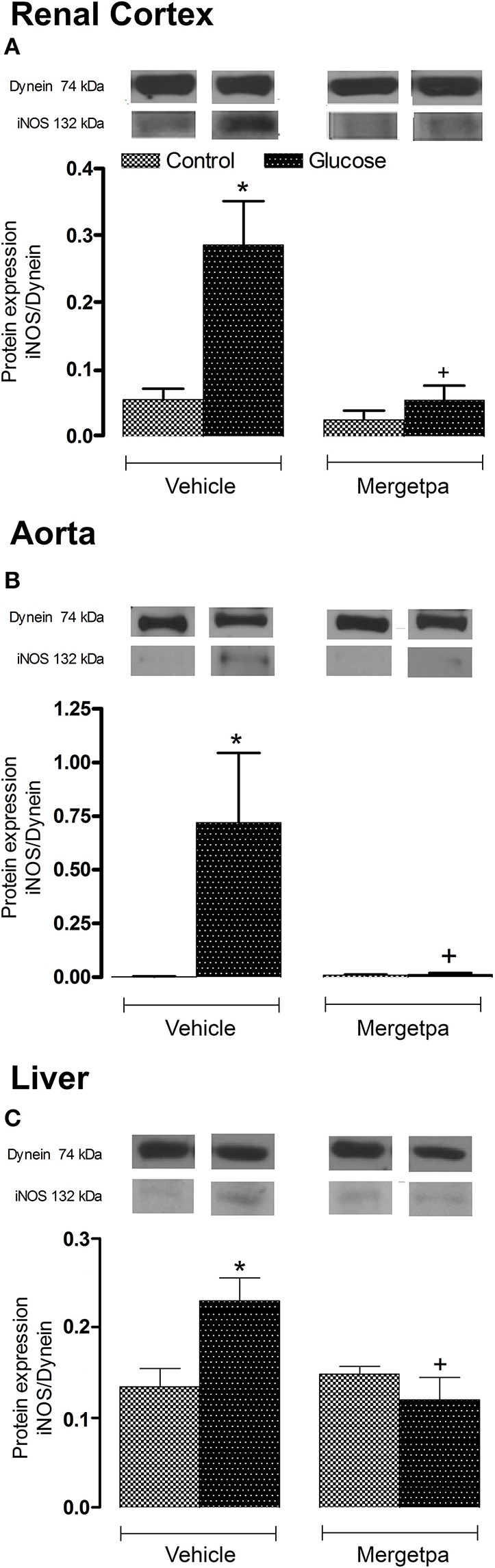
Treatment effect on iNOS expression. Effect of s.c. administered Mergetpa (1 mg.kg ^−1^ twice daily) for 7 days on iNOS expression in **(A)** renal cortex, **(B)** thoracic aorta, and **(C)** liver. The expression of iNOS was measured at the protein level by western blot and dynein was the loading control. Data are mean ± SEM obtained from six rats per group. ^*^P < 0.05 compared with control + vehicle; ^+^*P* < 0.05 compared with glucose + vehicle. Data in vehicle treated rats are from a previous study (Haddad and Couture, 2016) but performed simultaneously.

As iNOS is activated by B1R and contributes to insulin resistance, its expression was measured in parallel to B1R and CPM in the three tissues of glucose-fed rats. Data presented in Figures 6A–C show that the protein expression of iNOS was markedly and significantly enhanced (*P* < 0.05) in the renal cortex, thoracic aorta and liver of glucose-fed rats. The 1-week treatment with Mergetpa abolished the overexpression of iNOS in the three tissues of glucose-fed rats without affecting its basal expression in control rats (Figure 6).

### Effect of Mergetpa on IL-1β expression

**Figure 7:**
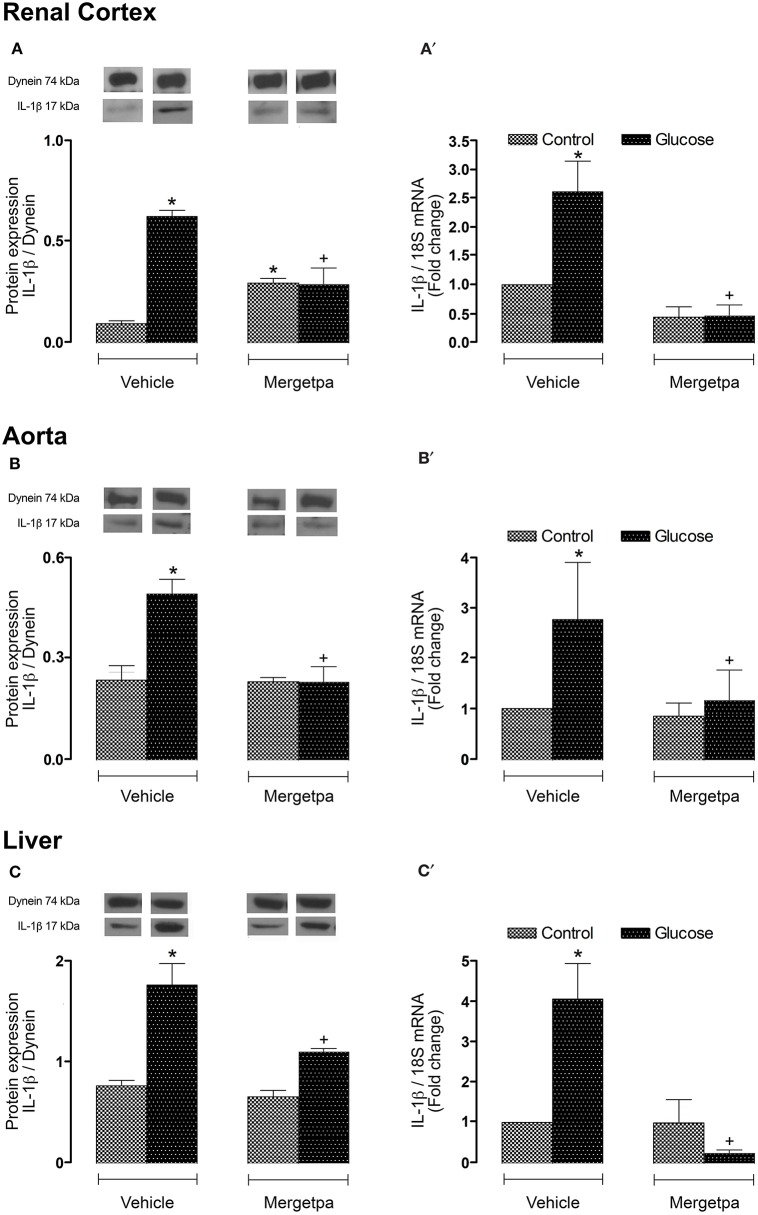
Treatment effect on IL-1β expression. Effect of s.c. administered Mergetpa (1 mg.kg ^−1^ twice daily) for 7 days on IL-1β expression in **(A,A')** renal cortex, **(B,B')** thoracic aorta and **(C,C')** liver. The expression of IL-1β was measured at the protein level by western blot **(A,B,C)** and at mRNA level by qRT-PCR **(A',B',C')**. Dynein was the loading control. Data are mean ± SEM obtained from six rats per group. ^*^P < 0.05 compared with control + vehicle; ^+^*P* < 0.05 compared with glucose + vehicle. Data in vehicle treated rats are from a previous study (Haddad and Couture, 2016) but performed simultaneously.

Because IL-1β is involved in the induction of B1R (Dias et al., 2010; Dias and Couture, 2012a; Couture et al., 2014; Haddad and Couture, 2016), it was of interest to measure the expression of this pro-inflammatory cytokine in the three studied tissues. Thus, it was found that IL-1β protein expression and IL-1β mRNA levels were significantly enhanced (*P* < 0.05) in renal cortex (Figures 7A,A'), thoracic aorta (Figures 7B,B') and liver (Figures 7C,C') of glucose-fed rats. The 1-week treatment with Mergetpa blocked completely IL-1β protein and mRNA overexpression in the three tissues of glucose-fed rats. While Mergetpa increased significantly IL-1β protein expression in the renal cortex of control rats, it had no significant impact in the other control tissues (Figure 7).

## Discussion

This study reinforces the detrimental role for kinin B1R in insulin resistance because the inhibition of the main enzymes (CPM/CPN) involved in the biosynthesis of the endogenous B1R agonists (des-Arg^9^-BK and Lys-des-Arg^9^-BK) improved hyperglycemia, hyperinsulinemia and the HOMA index of insulin resistance. This was associated with the correction of body weight gain and the reversal and normalization of the enhanced expression of several markers of inflammation (B1R, CPM, iNOS, and IL-1β in renal cortex, thoracic aorta and liver) and of the oxidative stress (enhanced production of superoxide anion in aorta and nitrotyrosine expression in aorta and renal cortex). Importantly, results obtained with Mergetpa are closely similar to those reported earlier in this model of insulin resistance after 1-week treatment with the B1R antagonist SSR240612 (Dias et al., 2010; Dias and Couture, 2012a,b) or with the selective inhibitor of iNOS 1,400 W (Haddad and Couture, 2016). It was then suggested that the activation of B1R leads to the formation of peroxynitrite upon post-translational activation of iNOS and NADPH oxidase. In turn, peroxynitrite exerts a positive feedback loop to enhance the expression of B1R. Blockade of iNOS prevented this vicious cycle and the pro-inflammatory effects of B1R (Haddad and Couture, 2016). Herein, we suggest that peroxynitrite can also exert a positive feedback loop to enhance the expression of CPM providing further endogenous agonists to activate B1R. This appears a feasible mechanism for B1R upregulation as B1R can be upregulated by its own agonist (Schanstra et al., 1998; Couture et al., 2014). Blockade of B1R *in vivo* with SSR240612 also reversed the upregulation of B1R in peripheral tissues in glucose-fed rats (Dias et al., 2010; Dias and Couture, 2012a). Thus, pharmacological blockade of B1R with SSR240612 or the inhibition of the generation of B1R endogenous agonists with Mergetpa yielded the same outcome by suppressing B1R agonists-induced upregulation of B1R and subsequently B1R downstream signaling on iNOS, NADPH oxidase and other markers of inflammation.

### Impact of Mergetpa on cardiometabolic targets

Our data are consistent with previous studies showing that B1R contributes to insulin resistance and obesity through a mechanism independent of leptin. Indeed, hyperleptinemia found in glucose-fed rats remained unaffected by the prolonged inhibition of B1R with SSR240612 and iNOS with 1,400 W while body weight gain was reversed by both treatments (Dias and Couture, 2012b; Haddad and Couture, 2016). Moreover, a similar 1-week treatment with SSR240612 in obese Zucker diabetic fatty rats (ZDF), a model of type 2 diabetes, reversed body weight gain without affecting hyperleptinemia (Talbot et al., 2016).

Mergetpa failed to reduce high blood pressure associated with high glucose feeding that is not in accord with the anti-hypertensive effect of SSR240612 reported in this model (Dias et al., 2010). This could be explained by the finding that cerebral but not peripheral B1R are involved in the maintenance of high blood pressure as documented in glucose-fed rats, spontaneously hypertensive rats and angiotensin II-induced hypertension using peptide and non-peptide B1R antagonists (Lungu et al., 2007; De Brito Gariépy et al., 2010). At this time, there is no information regarding the possibility that Mergetpa can pass the blood-brain barrier to inhibit brain kininase 1. Further studies will be needed to address this issue. Nevertheless, our data highlight a dissociation regarding the mechanisms underlying insulin resistance and hypertension as previously evidenced with the iNOS inhibitor (Haddad and Couture, 2016), and support the idea that peripheral B1R do not contribute to hypertension as reported in other rat models (De Brito Gariépy et al., 2010; Couture et al., 2014).

### Impact of Mergetpa on inflammatory markers

The suppression of the enhanced expression of B1R, CPM, iNOS, and IL-1β by Mergetpa was quite clear in the three studied tissues (renal cortex, thoracic aorta and liver) in glucose-fed rats, suggesting that the inflammatory process was not limited to the vasculature. In agreement with the present findings, 1-week blockade of B1R with SSR240612 reversed the overexpression of vascular and non-vascular B1R, iNOS, and IL-1β in glucose-fed rats (Dias et al., 2010; Dias and Couture, 2012a,b).

Chronic activation of B1R is likely to be amplified by the accumulation of des-Arg^9^-BK and Lys-des-Arg^9^-BK at the site of inflammation because the half-life of des-Arg^9^-BK is 4- to 12-fold longer than that of BK (Decarie et al., 1996). Upregulation of CPM may also account for the increasing endogenous levels of des-Arg^9^-kinin metabolites and the subsequent upregulation and activation of B1R in the inflammatory process linked to insulin resistance.

CPM is likely more important than CPN in the metabolic regulation of kinin activity since these vasoactive peptides are primarily considered local hormones (autacoids) playing autocrine and paracrine functions (Carretero and Scicli, 1991). Membrane-bound CPM has been shown to play a critical role in the generation of specific agonists in close proximity to the B1R (Zhang et al., 2013a), which is supported by our *in vivo* data using Mergetpa. However, one cannot exclude the possibility that Mergetpa blocks B1R signaling independently of the enzymatic generation of B1R agonists by CPM. Recent molecular studies in stably transfected cells have shown that bradykinin or kallidin binds to CPM (without generating agonist) to induce a conformational change in the enzyme that is transmitted to the B1R causing its activation. Mergetpa blocks this conformational crosstalk by preventing the binding of kinins to CPM (Zhang et al., 2013a). Importantly, the present study confirms the contribution of CPM and excludes the possibility that cathepsin X could intervene in the formation of B1R ligands (Nagler et al., 2010) or that dynorphin could be an endogenous activator of the B1R (Lai et al., 2006, 2008; Lee et al., 2014) in this pro-inflammatory model of insulin resistance.

### Limitations of the study

CPM is a largely distributed enzyme that cleaves C-terminal lysine or arginine from other peptides and proteins, including anaphylatoxins, chemokines, enkephalins and growth factors (Deiteren et al., 2009) that may question its specificity as a pharmacological target. While the regulatory role of CPM in kinins and B1R activity has been fairly well documented (Erdos and Sloane, 1962; Zhang et al., 2013a,b; Couture et al., 2014; Regoli and Gobeil, 2015), the impact of CPM on the physiological functions of other endogenous substrates, and particularly in insulin resistance, is still unknown and remains to be studied. In the present study, the inhibition of B1R-agonists formation by Mergetpa has not been directly demonstrated and is not an easy task *in vivo* due to the short life of these peptides, but the impact of CPM blockade is in accordance with the beneficial effects of B1R antagonism in insulin resistance and its associated oxidative stress and inflammatory mechanisms (Dias et al., 2010; Dias and Couture, 2012b; Haddad and Couture, 2016). Mergetpa had no or little significant effect in control rats, which is consistent with the virtual absence of B1R in control rats (Couture et al., 2014). Hence, findings suggest specificity of action of the drug on CPM and on kinins as the main substrates.

## Conclusion

Pharmacological blockade of kininase 1 (CPM) for 1-week provided similar beneficial effects to a corresponding treatment with a B1R antagonist in insulin resistance and peripheral inflammation induced by high glucose feeding. These findings have clinical relevance in the treatment of type 2 diabetes and its complications. Hence, inhibiting kininase I to lower B1R agonists generation may provide a novel therapeutic approach as alternative to B1R antagonists, and has the advantage to enhance the cardioprotective and anti-diabetic effects of the B2R (Regoli and Gobeil, 2015) by preventing the cleavage of the COOH-terminal-Arg residue on BK and kallidin. The development of a new generation of kininase 1 inhibitors for pharmacotherapy is appealing and would be an asset.

## Author contributions

YH performed the experiments, analyzed the data, made the figures and drafted the manuscript. YH, RC interpreted the data and revised critically the intellectual content. RC designed the study, supervised the work and wrote the final version of the manuscript. All authors agree to be accountable for the content of the work.

### Conflict of interest statement

The authors declare that the research was conducted in the absence of any commercial or financial relationships that could be construed as a potential conflict of interest. The reviewer LB and handling Editor declared their shared affiliation, and the handling Editor states that the process met the standards of a fair and objective review.

## Abbreviations

BK: bradykinin
B1R: bradykinin type 1 receptor
B2R: bradykinin type 2 receptor
CPM: carboxypeptidase M (membrane)
CPN: carboxypeptidase N (plasmatic)
GPCR: G-protein-coupled receptor
HOMA: homeostasis model assessment index
IL-1β: interleukin-1β
Mergetpa: DL-2-Mercaptomethyl-3-guanidinoethylthiopropanoic acid
NADPH: nicotinamide adenine dinucleotide phosphate
NO: nitric oxide
NOS: nitric oxide synthase
iNOS: inducible nitric oxide synthase
ONOO^−^: peroxynitrite
qRT-PCR: quantitative real-time polymerase chain reaction
O_2_^•−^: superoxide anion.
